# An efficient flat-surface collar-free grafting method for *Arabidopsis thaliana* seedlings

**DOI:** 10.1186/1746-4811-9-14

**Published:** 2013-05-04

**Authors:** Nayelli Marsch-Martínez, John Franken, Karla L Gonzalez-Aguilera, Stefan de Folter, Gerco Angenent, Elena R Alvarez-Buylla

**Affiliations:** 1 Instituto de Ecología, UNAM, México, DF, México; 2 Plant Research International, Wageningen University and Research Centre, Wageningen, The Netherlands; 3 Departamento de Biotecnología y Bioquímica, y Departamento de Ingeniería Genética, CINVESTAV-IPN, Unidad Irapuato; 4 Laboratorio Nacional de Genómica para la Biodiversidad, CINVESTAV-IPN, Irapuato, Gto, México; 5 Present address: 431 Koshland Hall, Plant and Microbial Biology, University of California, Berkeley, CA, 94720, USA

## Abstract

**Background:**

Grafting procedures are an excellent tool to study long range signalling processes within a plant. In the last decade, suitable flat-surface grafting procedures for young Arabidopsis seedlings using a collar to support the graft have been developed, allowing the study of long-range signals from a molecular perspective.

**Results:**

In the modification presented here, scion and stock are put together on the medium without supporting elements, while cotyledons are removed from the scion, resulting in increased grafting success that can reach up to 100%. At the same time, the protocol enables to process as many as 36 seedlings per hour, which combined with the high success percentage represents increased efficiency per time unit.

**Conclusions:**

Growing cotyledons usually push the scion and the rootstock away in the absence of a supporting element. Removing them at the grafting step greatly improved success rate and reduced post-grafting manipulations.

## Background

Grafting procedures have been commonly and successfully used in many plant species for agricultural purposes since ancient times, and are also an excellent tool to study long range signalling processes within plants. The ability to use chimaeric plants can be instrumental for addressing questions concerning non cell-autonomous processes and long-range signal movement within plants. Elegant classic grafting experiments in plants like pea (*Pisum sativum*), white mustard (*Sinapis alba)* and other species have enriched the understanding of processes such as flowering time and hormone signalling, among others. In the last decade, suitable grafting protocols have also been developed for the model plant *Arabidopsis thaliana*, allowing the study of long range signals from a molecular perspective. Using grafting approaches, deeper molecular knowledge has been gained about signalling events participating in different processes such as flowering [1-4], shoot branching [5], heavy metal detoxification [6], nutritional status [7], small RNA movement [7,8] and long distance nuclear silencing [9,10], among others.

Different methods to graft Arabidopsis have been devised, which allow grafting very young seedlings or more developed, older plants [3,6,11-15], or even a single organ [1]. Most of these protocols use plants grown in contained environments. Also, different grafting configurations have been achieved. Some examples of these configurations are: “root-shoot” – made by joining the hypocotyls of the rootstock and scion and having a root of a genotype and the shoot of another, “two shoot Y-graft” – consisting of a rootstock that maintains its shoot and in which a second shoot is grafted, having a root and one shoot of a genotype, and another shoot of a different genotype; and “shoot-shoot” – where the inflorescence stem of the scion is grafted to the inflorescence stem of the rootstock, having the root and the basal part of the shoot of a genotype, and the apical part of another genotype [6,11,12].

In grafting methods for both young seedlings and older plants, the use of a silicon collar was highly effective [11,12,14]. In this paper, we propose a flat-surface root-to-shoot grafting protocol that does not require a collar to support the graft and produces a high-success rate (that can reach up to 100%) for young seedlings (6-7 day old), while increasing the number of seedlings that are grafted per hour. The proposed method also reduces the growth of adventitious roots and simplifies handling of grafted seedlings.

## Grafting methods, results and discussion

The general grafting conditions and procedures were performed following the valuable recommendations suggested in previously reported protocols [12,15]. In this work we aimed to test whether cotyledon removal could improve grafting efficiency and success in young Arabidopsis seedlings where no collar was used as a graft support. The procedures and results of grafting seedlings with two, one or zero cotyledons grown in different conditions are described below. A comparison of the percentage of grafting success of the different experiments is shown in Table 1.

**Table 1:** Effect of growth conditions and cotyledon removal in grafting success

**Day length**	**Sugar**	**Age (days)**	**Cotyledon number**	**Event**	**Total grafted**	**Successful**	**Success %**	**Observations**
SD*	0.5%*	4	0	1	45	23	51	
		6*	0*	1	34	31	91	
				2	40	31	78	
				3	8	8	100	
				4	66	56	85	
				5	82	61	74	
				6	42	39	93	
				7	40	23	58	horizontal
			1	1	54	31	57	
			2	1	12	5	42	
				2	34	7	21	
				3	54	14	26	
				4	20	7	35	
				5	70	31	44	
				6	25	13	52	
				7	26	11	42	
		7	0	1	42	34	81	
		8	0	1	35	24	69	
	0%	6	0	1	30	9	30	no leaves
			1	1	42	16	38	
				2	31	9	29	horizontal
LD	0.5%	4	0	1	44	17	39	
				2	33	22	67	
		5	0	1	39	20	51	
		6	0	1	39	11	28	
	0%	5	0	1	38	25	66	
				2	25	9	36	
				3	23	7	30	no leaves

Seedlings were grown in different day length and sugar conditions and grafted at different ages with two, one or zero cotyledons, as indicated in the table rows. The total number of Arabidopsis plants grafted and the number and percentage of successful grafts out of them is presented. “Event” numbers indicate independent grafting sessions. In “Observations¨, “no leaves” means that no leaves were visible 14 days after grafting, “horizontal” means that the seedlings were grown in a plate placed horizontally in the growth chamber. Asterisks indicate the combination of conditions that produced the highest grafting efficiency. *SD*, Short days; *LD*, Long days.

### Genotypes and Growth conditions

#### Genotype

Grafting experiments were performed with wild type or *DR5::GUS Arabidopsis thaliana* seedlings of Col-0 ecotype.

#### Day length

Initially, plants were grown and allowed to recover from the grafting procedure in short day conditions (8 hrs. light, 16 hrs. darkness), in a Weiss or a Percival growth cabinet, at 21 degrees Celsius. After observing that the highest grafting success in this condition was obtained by removing both cotyledons, tests were also performed in long days (16 hrs. light, 8 hrs. darkness), varying the age of the seedlings and sucrose content of the medium (Table 1). From these comparisons, it appeared that a higher grafting success was obtained under short day conditions than under long day conditions, possibly due to the slower growth of plants that could cause movement of the aligned scion and rootstock, helping the recovery of successfully grafted plants.

#### Medium

Seedlings were grown on a basis of 0.5X Murashige & Skoog Basal Medium w/vitamins (M519 PhytoTechnology Laboratories) with 1% agar, and grafts were also allowed to recover in the same medium. Different sugar concentrations were used, as described below.

#### Sugar content

Different sucrose concentrations were tested. In preliminary tests using 0.2% and 0.5% sugar, growth and graft success were satisfactory, with 0.5% producing optimal grafting success. In contrast, plants grown in 1% sugar showed signs of stress and less efficient grafting results (data not shown). Therefore, tests to compare the cotyledon-less grafting success with and without sugar were performed comparing sucrose concentrations of 0% vs. 0.5%. At 0% sugar plants grew slowly, showed a slower recovery after grafting, and the graft success rate was lower than when using 0.5% sugar (Table 1). Short day 0% sugar plants were particularly slow in development, and no leaves larger than 1 mm could be observed when evaluated 10 days after grafting. Therefore, for cotyledon-less grafting, using medium supplemented with 0.5% sugar concentration produced optimal grafting success.

Brosnan and collaborators [10] mention the removal of one cotyledon for grafting when necessary, with the purpose of orientating properly a seedling. We evaluated the effect of the systematic removal of both cotyledons and compared to removing only one cotyledon in the grafting process. In 0% sugar, the removal of a single cotyledon resulted in greater leaf growth of grafted plants than when both cotyledons were removed, but the grafting success was much lower than removing both cotyledons of seedlings allowed to recover in medium with 0.5% sugar (Table 1).

#### Plate orientation

Plates were placed almost vertically, with a slight tilt (about 5 to 10 degrees). This was important because letting seedlings grow in plates placed horizontally can result in bent cotyledons that are difficult to align and therefore increase the time of the procedure and decrease grafting success (see Table 1, compare the grafting success of rows marked as “horizontal” in the column “observations”).

### Grafting procedure

#### Plant age

4 to 8 days-old seedlings were tested for grafting [12]. Table 1 shows a comparison of the grafting success obtained using seedlings of different ages grown under different conditions, with two, one or zero cotyledons. We obtained highest grafting efficiency when using 6 or 7 days-old seedlings (Table 1). Younger seedlings were more difficult to handle and longer times were required to process them, specially at the step of removing cotyledons with a single cut without damaging the meristem, and therefore the time required for the grafting was longer in comparison to using 6 and 7 days-old seedlings. On the other hand, leaves of 8 days old seedlings expanded sooner after grafting and promoted the movement of the scion and rootstock before their attachment was strong enough to endure it without separation. In any case, at any age used, when etiolated seedlings were grafted the success was reduced (data not shown).

#### Materials

Binocular stereoscope. The quality of the binocular microscope to observe cuttings, place scion and rootstock together and remove adventitious roots was important for the grafting success. In these experiments, a ZEISS STEMI 2000-C or STEMI SV6 S 1,0x, and LEICA MZ75 stereoscopes provided good results.

#### Cutting blade

Different blades were tested. The Swann medical surgical blade #11 and the GEM scientific single edge razor blade worked well. Many cuts can be made with a single blade, but it is important to replace it when it losses sharpness. The best results were obtained when a new blade was used for each grafting session (from 60 to about 120 grafted seedlings).

#### Cutting supports

Different cutting supports were tested:

**Figure 1:**
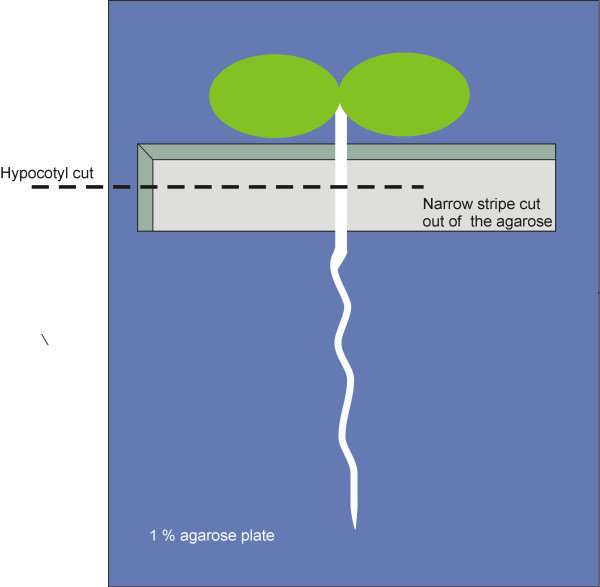
**Cutting surface used for Arabidopsis seedlings.** Arabidopsis seedlings are placed in a plate containing a thin layer of 1% agarose. The hypocotyl is positioned just above a “well” that has been left after removing a narrow block from the agarose layer. Cotyledons and root remain in the agarose layer, which avoids drying of the seedling while the hypocotyl can be cut dry and supported by a hard surface.

i. Empty plate with some water drops. The seedling to be cut was placed in the drop. Cutting was performed well, but the ends to be grafted became too wet, which made alignment difficult.

ii. 0.8 or 1% agar plates. Cutting the seedlings in agar plates produced variable grafting rates.

iii. Plates with a very thin layer of 1% agarose. The agarose layer was made as thin as possible (around 1 mm). Narrow agarose stripes were removed from the plate to make a long “well” in the agarose layer. Seedlings were placed on top and perpendicular to the ¨well¨ making sure that the top (upper hypocotyl containing the shoot apical meristem and cotyledons), and bottom (lower hypocotyl and root) of the seedling were resting in the agarose, while the middle part of hypocotyl was positioned above the “well” (Figure 1). Using this method, the cut in the hypocotyl was sharp and the seedlings did not bend as when placed in a soft surface. These were by far the best cutting conditions from the three tested.

#### Graft support

No support other than the medium itself was used to keep grafted plants together. They were placed together in the medium surface and allowed to heal in vertically oriented plates. However, when left in the scion, growing cotyledons usually pushed the scion and the stock away, reducing grafting success significantly. An important increase in grafting success without collars or another support around the junction was achieved when removing both cotyledons of the scion (Table 1).

### Cutting and union procedures

#### Working conditions

All steps, from preparing plates, sowing seeds, cutting and joining scions and rootstocks were carried out under sterile conditions in a horizontal flow cabinet. Utensils (blades, tweezers and blunt Pasteur pipettes) were flame-sterilized by dipping in ethanol before passing through the flame, and allowed to cool before cutting or manipulating the seedlings. When grafted seedlings were transferred to soil, the evaluation of grafting success was done in non-sterile conditions, and the transference to soil was done right after evaluation.

#### Cotyledon removal

**Figure 2:**
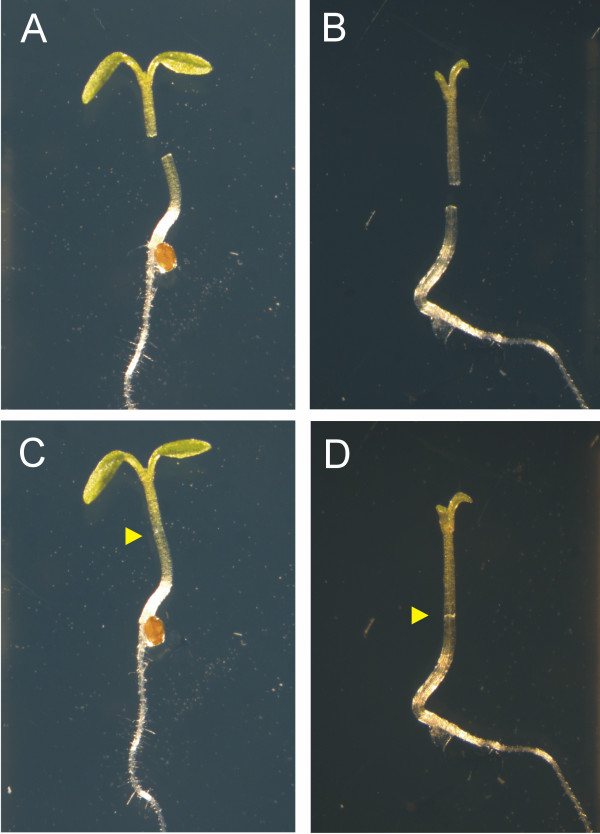
**Cut young seedlings for grafting. A** and **B**, seedlings ready to be grafted, with (**A**) and without (**B**) cotyledons. **C** and **D**, seedlings that have just been grafted, with (**C**) and without (**D**) cotyledons. Yellow arrowheads point to the union site of the grafted seedlings.

To avoid the pushing effect of growing cotyledons after grafting, we tested whether removing them increased grafting success (Figure 2). Before cutting, seedlings were transferred from the plates where they were grown to the thin layer agarose plates described in “cutting supports iii”. Both blunt forceps and Dumont tweezers (3C) were used to transfer them. When Dumont tweezers were used, their tips were placed under the cotyledons to lift them without closing or pinching.

Interestingly, cutting the cotyledons just before grafting greatly increased the grafting efficiency. Success rate ranged from 21% to 52% in different batches for plants grafted with cotyledons, and from 74% to 100% for plants grafted without cotyledons (Table 1). This result was unexpected. While the removal of a single cotyledon to attach the scion in its place is used for grafting other species such as melon [16], it has been reported that removing both cotyledons can be detrimental for graft healing, when grafting is performed in soil-grown plantlets of different species [17-19]. Interestingly, in this case, for grafting of Arabidopsis seedlings grown in medium, removing both cotyledons helped to increase grafting success rates. It may be that the effect of removing cotyledons is not detrimental in grafting success because seedlings can absorb nutrients from the medium, while higher success rates are attained probably because of a reduced movement of the grafting partners on the plate.

To make the process more time efficient, we placed more than one seedling in the cutting plate and cut all their cotyledons before proceeding with the next steps. We oriented the plate and cut first one of the cotyledons on the same side (all right or all left) of 3 to 6 seedlings and then turn the plate to cut the cotyledons on the other side. The hypocotyls were cut afterwards. This procedure allowed us to make up to 36 graftings per hour.

#### Hypocotyl cutting orientation

Hypocotyls were cut between the middle and the quarter above the middle, considering the SAM to be the top and the root to be the bottom (Figure 1). Transverse (90 degrees) or diagonal cuts were tested [12]. They both worked well, but partners with diagonal edges were more difficult to align, while transverse cuts allowed faster alignment and saved time. The hypocotyls were cut with a single sliding movement to reduce tissue damage.

#### Graft union

After cutting, it is important to join scion and rootstock as soon as possible, leaving enough space between each graft to grow. As indicated above, it is possible to cut a few (3 to 6) plants and then quickly proceed to arrange the desired graft combinations and join them. It is important to do this in a short time, with freshly cut pieces. We moved the cut pieces to the recovery plate, where the graft joining is done, just after cutting a hypocotyl, before cutting the next. For this, we used the tip of the scalpel to gently lift the scion at first. The scion naturally attaches to the wet metal surface of the scalpel and then is placed gently in the medium of the plate where the graft will recover. After moving the scion, the rootstock was also lifted in the same way with the tip of the scalpel and placed in the corresponding place of the plate.

In summary, we cut the first hypocotyl, moved the pieces to the “recovery” plate, cut the second hypocotyl and moved the pieces, and proceeded in the same way with the other seedlings. When all scions and rootstocks of the 3 to 6 seedling batch were in the “recovery” plate, they were joined. The final alignment of the scion and rootstock was done using two Pasteur pipettes. The tip of the pipettes was melted in fire and formed a blunt, smooth instrument that was used to gently push the pieces, sliding them in the medium and allowing fine movements to adjust both pieces without damaging them. For non-reciprocal graftings, where only one part of each plant will be used for the grafting, the procedure is the same, and only the part to be used (scion or rootstock) is transferred to the grafting plate. Either for reciprocal and non-reciprocal graftings, to avoid mistakes, we drew a line dividing the ¨cutting¨ plate in two and used one side (left or right) to cut a genotype, and the other side to cut the other genotype, marking each side accordingly. Similarly, when appropriate, we marked the right and left side of the plate where the graftings would recover so in one side graftings with scions of genotype 1 and rootstock of genotype 2 were placed, while in the other side genotype 2 scion and genotype 1 rootstock were located. It is important to avoid the presence of free liquid water in plates when joining grafts, because it complicates proper joining. If the recovery plates are too wet they can be left semi-open in the flow cabinet for some minutes before starting.

### Post-grafting handling

#### Removal of adventitious roots

**Figure 3:**
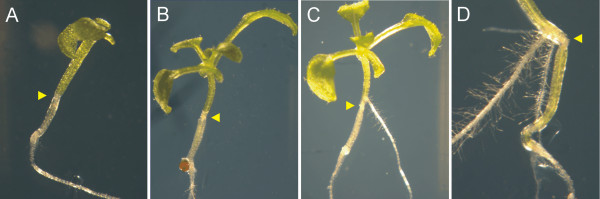
**Development of adventitious roots in grafted seedlings.** Grafted seedlings 11 (**A** &**D**), and 13 (**B** &**C**) days after grafting. Seedlings **A** and **B** were grafted without cotyledons and showed no adventitious root development. The few cotyledon-less seedlings that eventually develop adventitious roots are frequently those where the graft is compromised (**C**). Seedlings grafted with cotyledons showed adventitious root growth. (**D**) In this picture, a graft that did not take and produced adventitious roots is shown. Yellow arrowheads point to the union site of the grafted seedlings.

Some days after grafting, adventitious roots can appear above the graft junction and should be removed [12] (Figure 3). They were removed 10 to 14 days after grafting, with 10 days being optimal. Removing the cotyledons at the moment of grafting also reduced the formation of adventitious roots. This could be related to the fact that, as indicated by Turnbull and colleagues (2002), fast formation of the graft union diminished adventitious rooting. On the other hand, cotyledons could either provide nutrients, a signal or both, to support and stimulate adventitious root formation. In some cases, grafted plants devoid of cotyledons showing adventitious roots were those where grafting had not worked correctly, and could be directly discarded (Figure 3D).

#### Transfer to soil

One or two days after removing adventitious roots, the plants can be transplanted to soil. If necessary, the plants can be moved the same day, or left longer in the plate if the experiment requires it. It is important to continuously monitor the appearance of new adventitious roots above the graft junction (much easier in plates, harder in soil), at least when the plants are young. After the plants are transferred to soil, in short and long day conditions, the plants showed a normal wild type appearance.

### Grafting Protocol

All steps, except graft evaluation when plants will be transferred to soil, are performed under sterile conditions, in a flow cabinet and with flamed utensils. A detailed description of the handling procedures is written in ¨*working conditions*¨.

1. Surface sterilize Arabidopsis seeds with ethanol and bleach, and sow them under sterile conditions in plates containing ½ MS, 1% agar, and 0.5% sugar.

2. Leave the plates at 4°C for three nights, and transfer afterwards to short day conditions (8 hrs. light, 16 hrs. dark, 21°C). Plates should be almost vertically oriented (5 to 10 degrees).

3. After 6 days, transfer 1 to 6 seedlings to a “cutting” plate. This is a plate with a very thin layer of 1% agarose where a long narrow agarose block has been removed in order to make a long “well” in the agarose layer. Place the seedling with the cotyledons and root touching the agarose, while the middle of the hypocotyl is just above the “well” (see Figure 1). Cut the cotyledons of the seedlings at the lower half of the pedicel, taking care not to damage the apical meristem. Afterwards, prepare the grafting partners by making a transverse cut at the middle or a quarter above the middle of their hypocotyl. Make the cuts under a binocular microscope, using a sharp blade. Swann medical surgical blades #11 work well.

4. After cutting, place the parts to be grafted in a new plate with the same medium in which they were grown, and join the grafting partners as soon as possible. Use a binocular microscope and two blunt-flamed Pasteur pipettes to place both parts closely together, and check that they stay in place. Leave enough space between each grafted plant for new leaves to grow in the subsequent days.

5. Place plates with grafted plants back in a short day cabinet in a vertical orientation, and let them grow for 10 days.

6. After 10 to 14 days, evaluate graft junction and, if necessary, remove eventual adventitious roots formed in the scion (cutting cotyledons at the moment of grafting highly reduced formation of adventitious roots). Plants can be left in plates or moved to soil afterwards. It is important to carefully monitor as far as possible and remove adventitious roots formed above the graft junction.

### Examples of protocol applications

#### Basal-apical communication in an hormonal response

**Figure 4:**
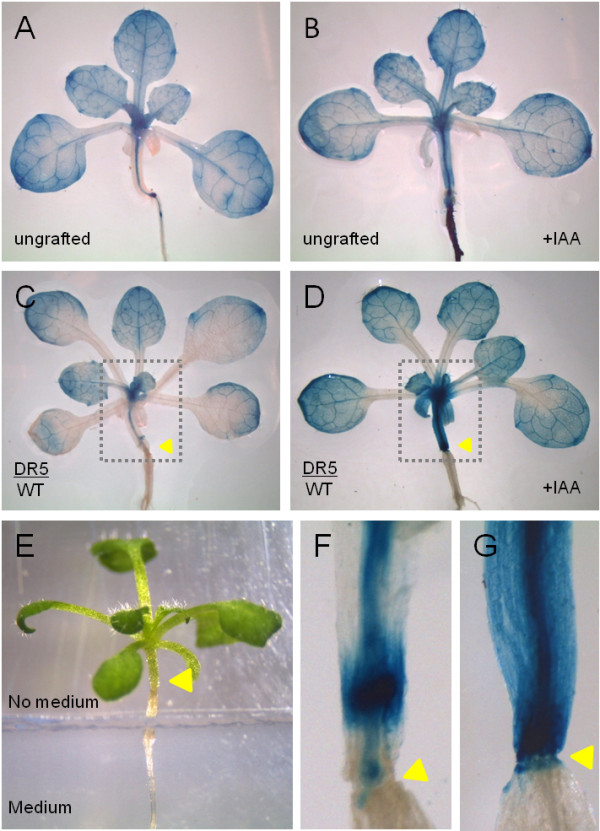
**Auxin response in *DR5::GUS*/WT grafted plants. A** and **B**) Ungrafted DR5::GUS plants. Cotyledons were removed at 6 dag and plants were transferred 7 days after to medium without IAA (**A**) or to medium supplemented with 5 μm IAA (**B**) for 3 days, and stained for GUS activity. Note the increased staining in the hypocotyl of the plant treated with IAA (**B**). **C** and **D**) Grafted DR5/WT plant after 3 days in medium without IAA (**C**) or medium supplemented with 5 μm IAA (**D**). Note that, as treated ungrafted plants, IAA treated grafted plants show increased staining, especially in the hypocotyl (dotted box) compared to untreated plants. **E**) In these experiments, only the rootstock was in contact with the medium and hormone. The picture shows a grafted plant that was transferred to a plate containing medium supplemented with 5 μm IAA; the agar was cut and the plants were placed in such a way that only the WT rootstock is in contact with the medium and the DR5 scion does not have any contact with it. **F** and **G**) Hypocotyl region of grafted DR5/WT plants in plates without IAA (**F**) or with 5 μm IAA (**G**), where increased staining in the hypocotyl of the IAA treated grafted plants compared to the untreated plants can be observed. Yellow arrowheads point to the union site of the grafted plants.

To test whether biological questions concerning the plant basal-apical communication-continuum could be answered using the method, we tested the hormonal response of grafts of reporter line scions with wild type rootstocks placed in contact with the hormone. Scions of the auxin marker line *DR5::GUS*[20] were grafted to wild type rootstocks following the protocol described above. 7 days after grafting, successful grafts were transferred to plates containing medium without or with 5 μM IAA (Figure 4). The agar medium was cut and the grafted plants placed in such a way that only the grafted wild type root and hypocotyl below the graft would be in direct contact with the medium, while the piece of hypocotyl of the scion could not touch it (Figure 4E). The plants were left on the medium for 72 hours under short day conditions, collected, and immersed in a GUS staining solution overnight at 37 degrees Celsius. After this, plants were cleared with ethanol and observed under a binocular microscope. Increased staining was observed in those *DR5::GUS* scions from grafted plants with IAA treated roots compared to untreated ones (Figure 4C and 4D). While untreated plants showed staining mainly at the vasculature (Figure 4F), treated plants showed staining in all the cells of the hypocotyl and increased staining at the shoot (Figure 4G). The same response was obtained when control ungrafted *DR5::GUS* were assayed (Figure 4A).

### The collar-free cotyledon-less grafting protocol works in other species

**Figure 5:**
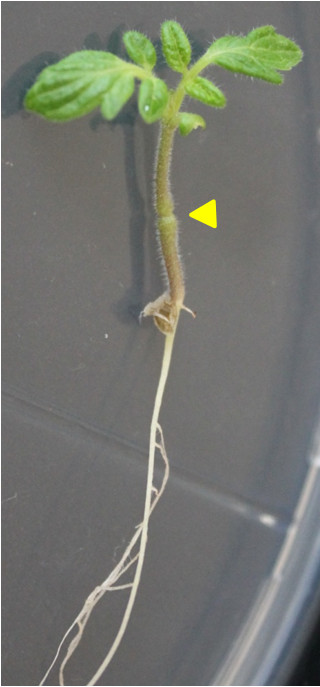
**Tomato grafted plant.** Tomato plant (*Solanum lycopersicum*, microtom) grafted without cotyledons 6 days after germination and grown under short day conditions for two weeks. No adventitious roots above the graft junction were observed. The yellow arrowhead point to the union site of the graft.

In order to test whether the method could be useful for other plant species, we tested this protocol in tomato seedlings grown in plates. For this, 6 days-old tomato (*Solanum lycopersicum* var. microtom) seedlings were grown in the same conditions as the Arabidopsis seedlings, with the exception that they were not subjected to the cold treatment prior to transfer to the growth chamber and germination occurred under long-day conditions. The method worked well and grafted seedlings developed a strong root-scion juncture as can be observed in Figure 5.

## Conclusions

In this paper we have shown that a collar-free grafting procedure in which both cotyledons are removed enables a greater number of successful grafted seedlings per unit of time than previously published methods in Arabidopsis. Grafting success of Arabidopsis seedlings depends on many factors. In the present protocol, that does not use supporting devices to keep stock and scion together, cutting cotyledons of the scion just before grafting improved greatly the grafting efficiency when seedlings were placed in growth medium. Some aspects of plant physiology and growth may be affected by removing cotyledons, but this method is useful for addressing many relevant questions, for example those concerning the root-shoot (and viceversa) communication mechanisms, including the transport of molecules among these two parts of the plant at different stages of plant development.

## Authors’ contributions

ERAB, NMM, and GA conceived the study. NMM, GA, and JF participated in its design. NMM, JF, SdF and KLGA performed the experiments and analyzed the results obtained. NMM and KLGA participated in preparing figures and table, NMM prepared the manuscript, and ERAB and GA revised it critically.
